# Purity Assessment of Aryltetralin Lactone Lignans by Quantitative ^1^H Nuclear Magnetic Resonance

**DOI:** 10.3390/molecules20069671

**Published:** 2015-05-26

**Authors:** Yan-Jun Sun, Yan-Li Zhang, Yu Wang, Jun-Min Wang, Xuan Zhao, Jian-Hong Gong, Wei Gao, Yan-Bin Guan

**Affiliations:** 1 Collaborative Innovation Center for Respiratory Disease Diagnosis, Treatment & Chinese Medicine Development of Henan Province, Henan University of Traditional Chinese Medicine, Zhengzhou 450046, China; E-Mails: zyl2013hnzy@163.com (Y.-L.Z.); wjmhnzz@163.com (J.-M.W.); lilas616@hotmail.com (X.Z.); gongjianhong2009@126.com (J.-H.G.); xiaojun801115@163.com (W.G.); 2 School of Pharmacy, Henan University of Traditional Chinese Medicine, Zhengzhou 450046, China; 3 School of Pharmacy, China Medical University, Shenyang 110122, China; E-Mail: xiaowangyu21@163.com

**Keywords:** qHNMR, HPLC-UV, purity, aryltetralin lactone lignan

## Abstract

In the present work, a quantitative ^1^H Nuclear Magnetic Resonance (qHNMR) was established for purity assessment of six aryltetralin lactone lignans. The validation of the method was carried out, including specificity, selectivity, linearity, accuracy, precision, and robustness. Several experimental parameters were optimized, including relaxation delay (D_1_), scan numbers (NS), and pulse angle. 1,4-Dinitrobenzene was used as internal standard (IS), and deuterated dimethyl sulfoxide (DMSO-*d*_6_) as the NMR solvent. The purities were calculated by the area ratios of H-2,6 from target analytes *vs.* aromatic protons from IS. Six aryltetralin lactone lignans (deoxypodophyllotoxin, podophyllotoxin, 4-demethylpodophyllotoxin, podophyllotoxin-7′-*O*-β-d-glucopyranoside, 4-demethylpodophyllotoxin-7′-*O*-β-d-glucopyranoside, and 6′′-acetyl-podophyllotoxin-7′-*O*-β-d-glucopyranoside) were analyzed. The analytic results of qHNMR were further validated by high performance liquid chromatography (HPLC). Therefore, the qHNMR method was a rapid, accurate, reliable tool for monitoring the purity of aryltetralin lactone lignans.

## 1. Introduction

Nuclear magnetic resonance spectroscopy (NMR), as a well-known analytical technique, has been routinely used for the structure elucidation of organic compounds, especially of newly synthesized and natural products. QHNMR was firstly published in 1963, for measuring the intra-molecular proton ratios in organic compounds [1]. In the past five decades, there has been growing interest in qHNMR. Experimental parameters, method validations, as well as major applications, were comprehensively discussed [2,3]. Due to the wide use of NMR spectrometers with high magnetic fields and the improvements in the probes, gradient shimming techniques, and efficient signal treatment methods, qHNMR has been ameliorated continually in sensitivity, resolution, and precision [4]. Numerous applications of qHNMR have been reported in the fields of phyto-preparations [5,6], agricultural products [7], natural products [8], disease diagnosis, environmental toxicity, metabolomics, pharmaceutics [2], and so on. It was confirmed that qHNMR was a reliable and accurate technique for quantitative analysis.

The chromatographic hyphenated techniques (such as LC-MS, GC-MS, and LC-UV) were placed in the main stream of pharmaceutical analysis. Meanwhile, the application of qHNMR for natural products was also described detailedly [9]. In comparison with chromatographic quantitative techniques, qHNMR has special advantages [9]: (i) The resonant signal intensity is directly proportional to the number of corresponding nuclei, which make it possible to quantify by the integral area ratio between the specific signal of the target analyte and that of IS; (ii) The chemical shift is related to the molecular structure, which ensures the selectivity of the qHNMR method. Simultaneously, the most comprehensive structural information from target analyte and impurities is reflected in the ^1^H-NMR spectra, including stereochemical details. However, in chromatographic quantitative techniques (such as HPLC-UV), only quantitative information can be provided; (iii) The analyzing time is relatively short. Sample preparation is simple, rapid, and without clean-up and derivatization; (iv) The technique is non-destructive, so samples can be applied for subsequent analyses and/or pharmacological investigations; (v) The outstanding advantage of qHNMR is that it can be applied in the purity estimation of organic compounds without using any specific reference standard. The qHNMR technique has been successfully applied to the purity test of organic compounds, especially complex natural products [3,9,10,11].

The aryltetralin lactone lignan occupies a unique position among natural products, and is found in the plants of genus *Sinopodophyllum*, *Podophyllum*, *Dysosma*, *Diphylleia* (Berberidaceae), *Linum* (Linaceae), Libocedrus (Cupressaceae), Bursera (Burseraceae), *etc.* [12]. Plants containing a high content of podophyllotoxin, such as *Sinopodophyllum emodi* and *Dysosma versipellis,* possess multiple biological activities, including anticancer, antioxidant, antivirus, antiradiation, *etc.* [13,14]. Our research team has long focused on chemical and pharmacological investigations of *Sinopodophyllum emodi*, in which aryltetralin lactone lignans are the representative and main bioactive constituents. Analytical methods described in literatures are mainly based on thin-layer chromatography, HPLC, and micellar electrokinetic chromatography [15,16]. Among those chromatographic quantitative techniques, HPLC with a UV detector has been widely used. However, some contaminations which lack UV absorbance cannot be detected. Most importantly, natural standard reference substances needed for the preparation of a calibration curve such as many aryltetralin lactone lignans are not available on the market. Thus, the purity evaluation of such natural products is vitally important for further pharmacological research and quality control of related herbal medicines.

## 2. Results and Discussion

QHNMR was developed and validated for the purity test of aryltetralin lactone lignans. 1,4-dinitrobenzene was used as internal standard. Six aryltetralin lactone lignans (deoxypodophyllotoxin, DPD; podophyllotoxin, PD; 4-demethylpodophyllotoxin, DMPD; podophyllotoxin-7′-*O*-β-d-glucopyranoside, PDG; 4-demethylpodophyllotoxin-7′-*O*-β-d-glucopyranoside, DMPDG; 6′′-acetyl-podophyllotoxin-7′-*O*-β-d-glucopyranoside, PDAG) were chosen to verify the qHNMR method. Their structures were shown in Table 1. The results acquired were further validated by HPLC-UV method.

**Table 1:** Chemical structures of aryltetralin lactone lignans tested. 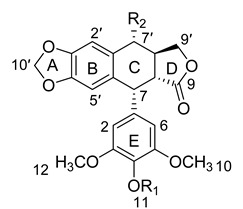

No.	Name	Abbreviation	R_1_	R_2_	Formula
1	deoxypodophyllotoxin	DPD	CH_3_	H	C_22_H_22_O_7_
2	podophyllotoxin	PD	CH_3_	OH	C_22_H_22_O_8_
3	4-demethylpodophyllotoxin	DMPD	H	OH	C_21_H_20_O_8_
4	podophyllotoxin-7′-*O*-β-d-glucopyranoside	PDG	CH_3_	β-d-glc	C_28_H_32_O_13_
5	4-demethylpodophyllotoxin-7′-*O*-β-d-glucopyranoside	DMPDG	H	β-d-glc	C_27_H_30_O_13_
6	6′′-acetyl-podophyllotoxin-7′-*O*-β-d-glucopyranoside	PDAG	CH_3_	6-acetyl-β-d-glc	C_30_H_34_O_14_

### 2.1. Method Validation

#### 2.1.1. Specificity and Selectivity

**Figure 1:**
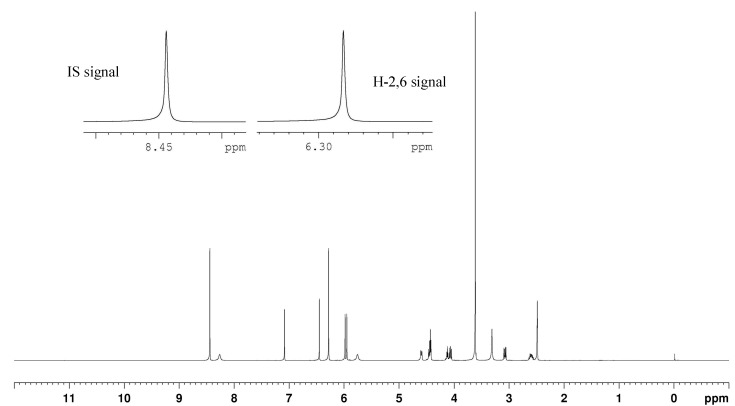
^1^H-NMR spectrum of 4-demethylpodophyllotoxin and 1,4-dinitrobenzene (IS) (500 MHz, DMSO-*d*_6_).

The specificity and selectivity of qHNMR improved greatly because it gave the structural information and content of target analytes and even impurities. The specificity and selectivity were evaluated through detection of possible interfering signals that were present in the sample solution. As can be seen from Figure 1, the baseline was straight and flat from δ 6.2 to δ 9.0, and the H-2,6 signals of target analyte were well separated from aromatic protons of IS. Meanwhile, the peak shapes of H-2,6 and IS protons were symmetrical. The S/N values of these two signals were above 1000. Therefore, the signals of H-2,6 can be used specifically for quantitative analysis.

#### 2.1.2. Linearity

**Figure 2:**
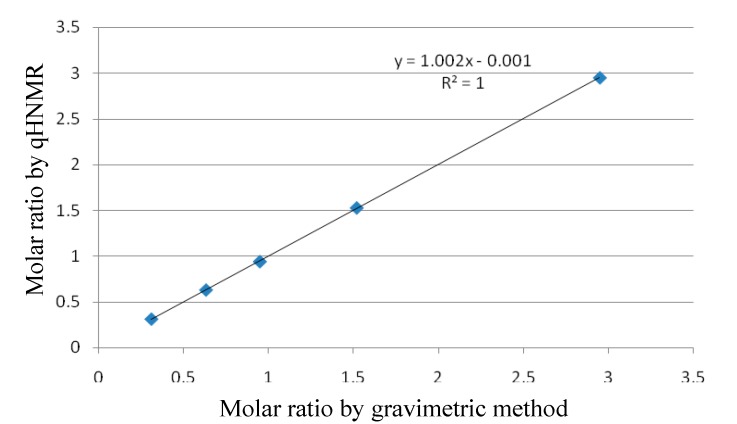
Linearity test of 4-demethylpodophyllotoxin: theoretical and experimental molar ratio of the standard solution.

In ^1^H-NMR spectra, the intensity of the resonant signal is directly proportional to the number of corresponding nuclei, so qHNMR is theoretically considered linear [17]. In order to test this experiment for linearity, a series of model solutions were prepared by dissolving the analytically weighed IS and 4-demethylpodophyllotoxin in DMSO-*d*_6_ in different molar ratios including 0.946, 0.6291, 0.3078, 1.516, and 2.947. For example, preparation of linearity samples was shown in Table 2. The calibration curve was subsequently plotted by the linear regression analysis of the experimentally determined molar ratios *vs.* the gravimetric reference values. As shown in Figure 2, clear linear regression (y = 1.002 x − 0.001) was obtained with a correlation coefficient of 1, indicating an excellent linear relation.

**Table 2:** Preparation of linearity samples and linearity test.

Weight of DMPD (mg)	Weight of IS (mg)	Gravimetric Molar Ratio	Molar Ratio by qHNMR
5.87	1.29	0.946	0.939
3.48	1.15	0.6291	0.6281
1.54	1.04	0.3078	0.3092
8.53	1.17	1.516	1.527
15.45	1.09	2.947	2.952

#### 2.1.3. Accuracy and Precision

Accuracy of a quantitative method is represented as the proximity relationship between a theoretical and experimental value [18]. In this study, accuracy was investigated by measuring the mean recovery and relative standard deviation (RSD). Three sets of recovery samples were analyzed by the qHNMR method. In the recovery range of 99.3%–100.9%, relative errors between the experimental and corresponding theoretical values were also less than 1%. As shown in Table S1, the average recovery was 100.2%, and the RSD value was 0.51%. Consequently, the results suggested the proposed method was accurate in the purity determination of aryltetralin lactone lignans.

Precision can be evaluated by RSD from the repeatability test, indicating the relative errors of replicate measurements obtained from the same homogenous sample [18]. To evaluate the intra-assay and intermediate precisions, twelve sample solutions were prepared independently, with approximately the same molar ratio of 4-demethylpodophyllotoxin and IS. The intra-assay precision was acquired from six repeated determinations. The intermediate precision was determined on an alternative NMR probe (a 5 mm PABBI 1H/D-BB inverse probe) by a different analyst on different days. As shown in Table S2, RSD values of intra-assay and intermediate precisions were found to be 0.32% and 0.36% respectively, indicating that the proposed method was highly reproducible and reliable.

#### 2.1.4. Stability

Stability testing is essential to determine the allowed time span between sample pretreatment and sample analysis. The same sample solution was assessed at 0, 8, 16, and 24 h after preparation, to access the solution stabilities. As shown in Table S3, the maximum % difference and RSD of the parallel detections were 0.5% and 0.34%, respectively. It was confirmed that the sample solution was stable up to 24 h at ambient temperature.

#### 2.1.5. Robustness

Robustness is interpreted as the reliability during normal routine usage. It is a requisite for quantitative analysis that suitably slight variations in the analytical parameters have no influence on measurement ability [18]. The robustness was evaluated by varying all key acquisition and data processing parameters independently, including acquisition time (AQ), relaxation delay (D_1_), spectral width (SW), scan numbers (NS), pulse angle (P_1_), and temperature. The influences of those parameters on the analysis results were examined with the same sample solution. Table 3 summarized all parameters examined and their variations. The optimal parameter values were denoted in bold. Within their evaluated ranges, most of the parameters, such as pulse angle, temperature, and spectral width, did not affect significantly the analysis results. For example, the purities of 98.03%, 98.03%, and 98.04% were obtained when pulse angle was fixed at 30°, 45°, and 90°, respectively. The purities of 97.97%, 97.97%, and 98.00% were achieved by temperature 293 K, 298 K, and 313 K, respectively. Acquisition time was investigated from 1.0 to 4.0 s at every 0.4 s. Every average integration of monitored signals was constant from 3.2 to 4.0 s. Integrations of monitored signals showed excellent precisions between 3.2 s and 4.0 s (0.38% for DMPD and 0.27% for IS, RSD). Therefore, the optimal acquisition time was 3.2 s. A 5% change of standard SW also did not alter quantification results. However, this was not the case for some parameters, such as relaxation delay (D_1_). An inappropriate value of D_1_ would cause an incomplete integral recovery and a quantification error. Herein, D_1_ was required to be at least 16 s, which will be discussed particularly in Section 2.5.1. later. For data processing, phasing, baseline correction, and integration were performed manually. All spectra were processed six times, and then average values were calculated. Above all, the quantitative results illustrated the robustness of the method.

**Table 3:** Summary of all examined parameters and their variation ranges.

Acquisition Parameters	Variation
Number of scans (NS)	8, **16**, 32, 64,128
Spectral width (SW, ppm)	10,15, **20**, 25, 30
Transmitter frequency offset (O_1_P, ppm)	4.5, **7.39**, 10.0
Relaxation delay (D_1_, s)	1, 2, 5, 10, 12, 14, **16**, 18, 20, 30, 50
Temperature (TE, K)	293, **298**, 313
Pulse length for excitation (P_1_, μs)	11.5, **11.78**, 12.5
Acquisition time (AQ, s)	1.0, 1.4, 1.8, 2.2, 2.8, **3.2**, 3.6, 4.0

### 2.2. Selection of Deuterated Solvent

A suitable deuterated solvent for qHNMR analysis is selected according to two important criteria [19]: (i) The analyte should be freely soluble in the solvent at the requisite concentration; (ii) The target resonances of analytes should have no interference from the solvent signal. In this study, pyridine-d_5_, CD_3_OD, DMSO-*d*_6_, D_2_O, and CDCl_3_ were investigated. The aglucones and glucosides of aryltetralin lactone lignans were practically insoluble in D_2_O and CDCl_3_, respectively, so the two solvents were eliminated. The solvent signal of pyridine-d_5_ imperfectly interfered with the target signals (δ 8.44) from the IS. The target analyte (deoxypodophyllotoxin) was sparingly soluble in CD_3_OD. Fortunately, DMSO-*d*_6_ met the above-mentioned criteria and was chosen as the deuterated solvent for qHNMR testing of aryltetralin lactone lignans.

### 2.3. Selection of Internal Standard

So far, various internal standards have been discussed and used in qHNMR [20]. Maleic acid, fumaric acid, tetramethylsilane (TMS), sodium acetate, and 1, 4-dinitrobenzene were thoroughly investigated for chemical-shift referencing and quantitative analysis. According to spectral characteristics of aryltetralin lactone lignans, an optimal IS signal may be assigned at the chemical shift ranges of δ 0–1.5, 1.7–2.4, 5.5–5.7, 7.5–8.1, or over 8.3. TMS was soluble in DMSO-*d*_6_, showing an ideal quantitative signal (δ 0, s). However, as a highly volatile liquid, it was unsuitable for quantitative analysis. Maleic acid with stable crystalline phase was weighed easily, and yet its sharp singlet at around δ 6.0 overlapped with methylenedioxy group proton signals of target analytes. Although its carboxylic acid proton signal at about δ 11.0 was free of any other signal interferences, the chemical shifts and integral areas varied slightly from sample to sample. Unfortunately, for fumaric acid, its case was similar to maleic acid. The ^1^H-NMR spectrum of sodium acetate gave a methyl singlet at around δ 1.8, which avoided overlapping with signals of target analytes. Nevertheless, it was partially soluble in DMSO-*d*_6_. 1,4-dinitrobenzene was chosen as internal standard (IS), mainly because of a sharp singlet that did not overlap with the signals of the target analytes. Besides, it presented other advantages: low cost, high purity, non-volatile, non-reactive, easy availability, long term stable, easily weighable, soluble in the selected NMR solvent (DMSO-*d*_6_), and a very simple ^1^H-NMR spectrum consisting of a sharp four proton singlet. Consequently, 1,4-dinitrobenzene was selected as internal standard.

### 2.4. Selection of the Quantification Signals from the Target Analytes

**Figure 3:**
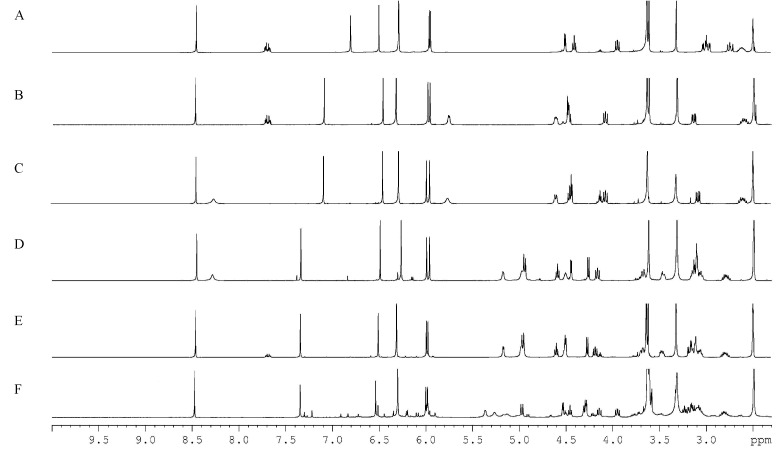
^1^H-NMR spectra of six aryltetralin lactone lignans (500 MHz, DMSO-*d*_6_): (**A**) DPD; (**B**) PD; (**C**) DMPD; (**D**) DMPDG; (**E**) PDG; (**F**) PDAG. 1,4-Dinitrobenzene (IS) signal was a singlet at δ 8.44.

Based on the quantification signals of IS, the quantification signals from the target analytes should meet the criteria given below [21]: (i) The signal should be separated clearly from any other signals; (ii) Its signal is situated near to IS signals, in order to reduce the influences of phase and baseline corrections; (iii) The singlet signal is superior to doublet or multiplet for the sake of a better signal-to-noise ratio. The ^1^H-NMR spectroscopic data of the analytes were shown in Table 4, and the ^1^H-NMR spectra for the target analytes with IS were shown in Figure 3. In ^1^H-NMR spectra of the target analytes, the highest signal level at δ *ca.* 6.30 was chosen as the quantification signal within the aromatic proton region. This signal, which was representative of two hydrogens, showed a good intensity, so that the sensitivity of the method increased. The quantification signals for the target analytes and IS do not suffer any interference of other possible protons. Thus, the quantification signals were selected as H-2,6 from the target analytes.

**Table 4:** ^1^H-NMR data for six aryltetralin lactone lignans (500 MHz, DMSO-*d*_6_, δ H (*J* in Hz)).

No.	DPD	PD	DMPD	DMPDG	PDG	PDAG
H-2	6.30, s	6.32, s	6.28, s	6.26, s	6.31, s	6.30, s
H-6	6.30, s	6.32, s	6.28, s	6.26, s	6.31, s	6.30, s
H-7	4.52, d, 5.0	4.48, d, 4.9	4.43, d, 5.2	4.45, d, 4.8	4.50, d, 4.8	4.53, d, 4.8
H-8	2.97, dd, 5.0, 13.9	3.14, dd, 5.1, 14.2	3.08, dd, 5.1, 14.2	3.12, m	3.18, dd, 4.8, 14.6	3.18, dd, 4.8, 14.5
H-10	3.63, s	3.63, s	3.62, s	3.62, s	3.63, s	3.62, s
H-11	3.60, s	3.61, s			3.61, s	3.61, s
H-12	3.63, s	3.63, s	3.62, s	3.62, s	3.63, s	3.62, s
H-2′	6.80 , s	7.09 , s	7.09 , s	7.34 , s	7.34 , s	7.35, s
H-5′	6.50, s	6.46, s	6.45, s	6.49, s	6.51, s	6.54, s
H-7′	3.01, dd, 5.2, 15.8; 2.73, dd, 11.7, 15.8	4.61, dd, 6.0, 9.1	4.60, d, 9.6	4.94, d, 9.7	4.96, d, 9.8	4.96, d, 10.2
H-8′	2.61, m	2.59, m	2.60, m	2.80, m	2.79, m	2.81, m
H-9′	4.41, t, 7.6; 3.94, dd, 8.3, 10.5	4.47, dd, 8.0, 8.6; 4.08, dd, 8.6, 10.5	4.45, dd, 7.8, 8.6; 4.07, dd, 8.6, 10.5	4.59, dd, 7.6, 8.6; 4.17, dd, 8.6, 10.4	4.60, dd, 7.7, 8.7; 4.18, dd, 8.7, 10.3	4.46, dd, 7.6, 8.6; 4.18, dd, 8.6, 10.2
H-10′	5.96, s; 5.94, s	5.98, s; 5.96, s	5.98, d, 0.8; 5.95, d, 0.8	5.99, s; 5.96, s	5.99, s; 5.97, s	6.00, s; 5.98, s
H-1′′				4.26, d,7.7	4.27, d,7.7	4.28, d,7.5
H-2′′				3.04–3.17, m	3.04–3.18, m	3.05–3.25, m
H-3′′				3.04–3.17, m	3.04–3.18, m	3.05–3.25, m
H-4′′				3.04–3.17, m	3.04–3.18, m	3.05–3.25, m
H-5′′				3.04–3.17, m	3.04–3.18, m	3.05–3.25, m
H-6′′				3.68, m; 3.47, m	3.68, m; 3.46, m	3.95, dd, 8.4, 11.8; 4.31, dd, 1.7, 11.8
COCH_3_						1.63, s

### 2.5. Optimization of Experiment Parameters

#### 2.5.1. Pulse Angle and Relaxation Delay

Pulse angle is interrelated with relaxation delay (D_1_). As the most important analytic parameters, the two parameters would directly affect the accuracy of qHNMR. Normally, a 90° pulse angle yielded maximum signal intensity. However, D_1_ was set to be more than five times of the longest longitudinal relaxation time (T_1_) of the quantification signal [2]. Thus, in order to reduce the total measuring time, a lower pulse angle was required for the complete resonance relaxation. As a 30° pulse angle was used, and D_1_ should be more than 7/3 times T_1_ [19]. T_1_s of target analytes and IS were briefly estimated by the inversion recovery experiment. The IS protons had relatively long (about 6.7 s), and H-2,6 from target analytes had short T_1_s (1.5–1.8 s). Therefore, D_1_ should be set as more than 16 s corresponding to 7/3 T_1_, which was further investigated by the effect of D_1_ on the peak area ratio of 4-demethypodophyllotoxin *vs.* IS. ^1^H-NMR spectra of the standard sample were accumulated with different D_1_ of 1, 2, 5, 10, 12, 14, 16, 18, 20, 30, and 50 s. The peak area ratio of 4-demethypodophyllotoxin *vs.* IS decreased with D_1_ increasing from 1 to 16 s, and then became constant. Therefore, D_1_ of 16 s was sufficient to make integrals of target quantification signals recover completely. It was set as 16 s in subsequent experiments.

#### 2.5.2. Sensitivity, Scan Number and Signal-to-Noise Ratio

NMR spectroscopy is known to be not very sensitive [17]. However, in this experiment sensitivity was sufficient. First, the monitored signals of target analytes and IS were separated completely. Secondly, the sensitivity can be increased by high-field spectrometer (500 MHz), gradient shimming technique, and cryoprobe. Thirdly, the sensitivity can also be enhanced by increasing the number of scans. The sensitivity is often indicated by signal-to-noise ratio (S/N). To obtain the precise quantitative results, S/N should be over 250:1 in qHNMR [2]. S/N was influenced by many factors, including number of spins in the system, gyromagnetic ratio of the detected and excited nucleus, number of scans, external magnetic field, transverse relaxation time, sample temperature, and the type of probe. Most of influencing factors were fixed at the discretion of the nuclide involved and NMR instrument configuration [2]. When other parameters were kept constant, the number of scans can be increased to achieve a satisfactory S/N. According to the S/N value, the scan number was set as 16 in this experiment.

#### 2.5.3. Phase Correction, Baseline Correction and Integration

Proper signal phasing is required to achieve accurate intensity measurement. Inaccurate phase correction may significantly result in the error of peak-ratio between the two signals. Consequently, the absolute or relative concentration of target analytes is also erroneous. An uneven baseline and an inaccurate integral region introduce a significant error of integrated peak area [2]. The phase correction and baseline correction were manually performed with great care. The range of the integral region greatly affects the quantitative accuracy [2]. In order to cover more than 99% of the total peak area, the integral region should be extended to a frequency range of 64 times the full width at half signal height [22]. Start and end points of the peak area integration were regulated manually. The quantification signals were integrated six times, and then the testing values were averaged to reduce the analytic errors that could arise during integration.

#### 2.5.4. Analysis Results by qHNMR and HPLC

**Figure 4:**
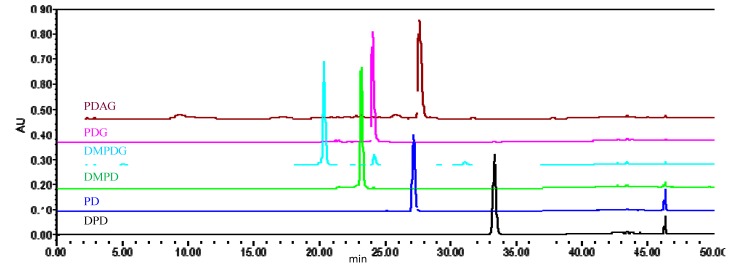
Representative HPLC chromatograms of six aryltetralin lactone lignans.

Purities of aryltetralin lactone lignans were also investigated by HPLC-UV technique. Representative chromatograms and analysis reports were shown in Figure 4, Figures S16–S21, and Tables S4–S9, respectively. Optimal HPLC analytical conditions were required to ensure the baseline separation of the target analytes and impurities. Thus, detection wavelength, different elution mode, flow rate, and column temperature were optimized systematically. With detection wavelength of 254 nm, some impurities were not responsive. Using the isocratic mobile phase of methanol/water (45%, 55%, or 65%), those target analytes cannot be differentiated by the retention time. The screening results indicated the gradient elution of methanol (A)/water(*v*/*v*) (B) (35%–65% A at 0–35 min, 65%–100% A at 35–50 min.) gave excellent separation of the target analytes and impurities, when the flow rate, column temperature and detection wavelength were at 1.0 mL·min^−1^, 25 °C, and 210 nm.

As summarized in Table 5, the obtained purity values of DPD, PD, DMPDG, and PDAG were less than 95%. The results investigated by the HPLC-UV analytic system were in good agreement with those assessed by the qHNMR method, with differences of less than 1.3%. The purity value is slightly higher by HPLC (PDAG, PDG, DMPDG, PD, and DPD), and for DMPD it is the opposite. The discrepancies were acceptable, when the measurement uncertainties and systematic errors of those two methods were considered. The possible reasons behind these differences were as follows: Firstly, the moisture absorption property of target analytes may be an influencing factor. For glucosides (DMPDG, PDG and, PDAG), the analysis results of qHNMR were smaller than their corresponding results for HPLC, which could be explained by absorbing moisture in the course of sample pretreatment. The trace amounts of conjugated water in the tested samples had no response in the UV detector. However, it can be detected sensitively by the ^1^H-NMR spectrum. Secondly, the HPLC-UV is not ideal in this case as the impurities do not have a chromophore, or do have a chromophore which is absorbing at a different wavelength. The ultraviolet absorption coefficients between unknown impurities and target analytes may vary dramatically. Accordingly, the inaccurate purity values were obtained probably by the HPLC-UV-area normalization method. Since the intensity of an evoked signal is directly proportional to the number of corresponding nuclei in the ^1^H-NMR spectra, whether impurities or target analytes, the peak area under specific signals can be used for quantitative research. Only from this perspective, were the analytic results more accurate by the qHNMR method. However, the validated qHNMR method was inappropriate for quantitative analysis of natural product mixtures such as *Sinopodophyllum emodi* extracts. The H-2,6 signals from different aryltetralin lactone lignans would overlap each other greatly, making it impossible to identify a given H-2,6 signal and to integrate accurately. The impurity information was reflected by the HPLC-UV method and ^1^H-NMR spectrum. Taking PDAG for example, there were some impurity peaks at 9.314, 17.156, 23.231, 25.722, 43.424, and 46.372 min in the HPLC chromatogram (Figure S16). Based on ^1^H-NMR spectra (Figures S3–S5), impurity signals were assigned at δ 7.30–6.72, 6.54–6.35, 6.21–6.08, 5.99, 5.98, 5.96, 5.90, 4.92–4.66, 4.51, 4.22–4.20, 2.02, 1.95, 1.22, and 1.13. The difference of purity measured by qHNMR and HPLC-UV was 1.26%, which may be explained mainly by the ultraviolet absorption coefficients of the impurities and target analytes. Thirdly, the subjective post-treatment of the NMR data could cause very small errors. The corrections of phase, baseline, and the choice of integral interval were performed manually. Fourthly, during the sample preparations, small deviations in weighing and volume calibrating gave rise to slight discrepancies in theoretical and experimental concentrations. The statistical consistency between the two independently determined purity values demonstrated that the qHNMR was a reliable and accurate technique for the purity assessment of natural products, especially aryltetralin lactone lignans.

**Table 5:** The purities of target analytes obtained by qHNMR and HPLC-UV.

Sample	qHNMR	HPLC-UV	Difference (%)
	Purity (%)	Purity (%)	
DPD	90.15 ± 0.27	90.78 ± 0.16	0.63
PD	89.53 ± 0.31	90.14 ± 0.29	0.61
DMPD	97.36 ± 0.42	96.51 ± 0.36	0.85
DMPDG	93.56 ± 0.37	94.61 ± 0.44	1.05
PDG	95.84 ± 0.26	96.72 ± 0.35	0.88
PDAG	90.38 ± 0.41	91.64 ± 0.52	1.26

## 3. Experimental Section

### 3.1. Reagents and Standards

#### 3.1.1. Testing Compounds

Deoxypodophyllotoxin (CAS No., 19186-35-7), podophyllotoxin (CAS No., 518-28-5), 4-demethylpodophyllotoxin (CAS No., 40505-27-9), podophyllotoxin-7′-*O*-β-d-glucopyranoside (CAS No., 16481-54-2), 4-demethylpodophyllotoxin-7′-*O*-β-d-glucopyranoside (CAS No., 40505-30-4), and 6′′-acetyl-podophyllotoxin-7′-*O*-β-d-glucopyranoside (CAS No., 917085-34-8). All the above listed compounds were isolated from the roots and rhizomes of *Sinopodophyllum emodi*. Their structures and stereochemistry were elucidated on the basis of spectroscopic evidence and circular dichroism (CD) method [12]. Testing compounds were stored at −23 °C and allowed to equilibrate in a desiccator (room temperature and reduced pressure) for 24 h before being tested, in order to prevent the formation of condensed water.

#### 3.1.2. Validation Standard and Internal Standard

Validation standard, 4-demethylpodophyllotoxin (purity, 98.0%), was obtained from Qingze Corporation of Nanjing (Nanjing, China). The internal standard (IS), 1, 4-dinitrobenzene (purity, 99.0%), was purchased from Tokyo Chemical Industry Co., Ltd. (TCI, Tokyo, Japan).

#### 3.1.3. Reagents

Methanol of HPLC grade (used as mobile phase) was purchased from Siyou Biology Medical Tech Co. Ltd. (Tianjin, China). Water was purified by means of a water purifier (18.2 MΩ) (Wanjie Water Treatment Equipment Co. Ltd., Hangzhou, China). Dimethyl sulfoxide-*d*_6_ (D, 99.9%) was obtained from Cambridge Isotope Laboratories, Inc. (Andover, MA, USA).

### 3.2. Instrument

^1^H-NMR spectra were recorded on a Bruker Avance-500 MHz NMR spectrometer (Bruker Biospin, Bremen, Germany), equipped with a QCI-F CryoProbe. All spectra were processed using Bruker’s Topspin software (version 3.0, Bruker Biospin, Bremen, Germany). The analytical HPLC data were measured on a Waters Alliance 2489 separations module equipped with a Waters 2695 UV/visible detector (Waters Co., Milford, CT, USA) and Empower pro data handling system (Waters Co., Milford, CT, USA). All samples were weighed on a Mettler 5 digit balance of ±10 µg.

### 3.3. Sample Preparations

#### 3.3.1. Standard qHNMR Samples for Method Validation

Standard qHNMR samples were prepared in the following protocol: About 1.5–15.5 mg of 4-demethylpodophyllotoxin and IS (*ca.* 1 mg) were accurately weighed into a 5 mL glass sample tube, and dissolved in 0.5 mL of DMSO-*d*_6_, bearing the different molar ratio of 4-demethylpodophyllotoxin *vs.* IS. The sample solutions were vortexed for 30 s, and transferred into NMR tubes prior to analysis.

#### 3.3.2. Testing qHNMR Sample

About 10 mg of IS was accurately weighed and dissolved in 1.0 mL of DMSO-*d*_6_, to produce an IS solution of ca.10.0 mg/mL. Approximately, 30–45 mg of target analytes were accurately weighed and dissolved in 1.0 mL of DMSO-*d*_6_, to produce the analyte solution of *ca.* 30.0–45.0 mg/mL. Then 100 μL of the IS solution, 200 μL of the analyte solution, and 200 μL DMSO-*d*_6_ were transferred into a 5 mL glass sample tube. The final solution (*ca.* 500 μL) was vortexed for 30 s and transferred into a 5 mm NMR tube. All the samples were made in triplicate.

### 3.4. Methods

#### 3.4.1. qHNMR Method

^1^H-NMR spectra were recorded at a central frequency of 500.19 MHz on a Bruker Avance-500 NMR spectrometer. The sample was not spun. Magnetic field homogeneity was performed by gradient-shimming, so that the full width at half IS signal height was adjusted to less than 1.5 Hz. Each sample was analyzed with the following parameters: a 30° pulse length (P_1_) of 11.78 μs, a spectral width (SW) of 10003.578 Hz, an acquisition time (AQ) of 3.2 s, scan number of 16, and a relaxation delay (D_1_) of 16.0 s. The temperature was set at 298 K. Free induction decays (FIDs) were processed with line broadening (LB) of 0.3 Hz prior to Fourier transformation. Preliminary data processing was achieved by Bruker software, version 3.0. Referenced to the IS residual signal at δ 8.44, the resulting spectra were manually phased, and then baseline correction was performed. For quantitative analysis, the resonant signals were integrated manually in appropriate regions (without ^13^C satellite signal, as possible). The signals of H-2,6 (δ *ca.* 6.30, s) from target analytes and the aromatic proton signal (δ 8.44, s) of IS were used for quantification Figure 4). In order to reduce the analytic errors from phase corrections and integrations, the mean integral values were obtained after all the spectra were processed six times. The purity of target analyte (*X*) was calculated according to the following formula [17]:

$$P_{X}=\frac{I_{X}}{I_{Std}}\times \frac{N_{Std}}{N_{X}}\times \frac{M_{X}}{M_{Std}}\times \frac{m_{Std}}{m_{X}}\times P_{Std}$$

where *I*, *N*, *M*, *m* and *P* are integral area, number of nuclei, molar mass, gravimetric weight, and purity of analyte (*X*) and IS (*Std*), respectively.

#### 3.4.2. HPLC-UV Method

Sample solutions for HPLC-UV analysis were prepared with methanol at a concentration of 0.2–0.5 mg/mL, and filtered through a 0.45 μm nylon filter (Jinteng Laboratory Instrument Co. Ltd., Tianjin, China). Analyses were accomplished on a YMC-Pack ODS A column (5 μm, 250 mm × 4.6 mm, YMC Co. Ltd., Kyoto, Japan) at 25 °C. Methanol (A)/water (v/v) (B) was used as the mobile phase in gradient elution mode as follows: 35%–65% A at 0–35 min, 65%–100% A at 35–50 min. The flow rate of the mobile phase was 1.0 mL/min^−1^. The target analytes were monitored at 210 nm by a UV/visible detector. The injection volume was 10 μL, and the running time was 50 min. The retention times of DPD, PD, DMPD, DMPDG, PDG, and PDAG were 33.368, 27.136, 23.204, 20.331, 24.069, and 27.632 min, respectively. Based on the peak area normalized to all observed HPLC peak areas, the purity of each target analyte was determined.

## 4. Conclusions

Purity assessment of natural products is particularly significant in the course of natural medicine discovery. Whenever chemistry is inextricably bound up with biological activity, trace contaminants of strong activity can result in false conclusions [23]. A qHNMR method was developed for the purity evaluation of six aryltetralin lactone lignans. 1,4-Dinitrobenzene was chosen as internal standard, and DMSO-*d*_6_ as the NMR solvent. Peak area ratios of H-2,6 signals (δ *ca.* 6.30, s) from target analytes *vs.* aromatic protons signal (δ 8.44, s) from IS were used for purity calculation. Crucial experimental parameters contributing to improvements in the analysis quality were carefully adjusted. Pulse angle, D_1_, and NS were set as 30°, 16 s, and 16 respectively. Method validation demonstrated that the established qHNMR method has excellent selectivity, linearity, accuracy, precision, stability, and robustness in the assessed molar-ratio range, and completely met the requirements of quantitative analysis. In addition, similar results were obtained by the HPLC-UV analytic system and the qHNMR method. However, qHNMR depends upon molecule structural complexity rather than physical properties, and is especially suitable for quantitative analysis of complex natural products with similar physical properties. Consequently, the qHNMR method is an important and reliable alternative to the conventional HPLC method.

## Supplementary Materials

Supplementary materials can be accessed at: http://www.mdpi.com/1420-3049/20/06/9671/s1.
