# Supplementary material for: Purity Assessment of Aryltetralin Lactone Lignans by Quantitative 1H Nuclear Magnetic Resonance
Source: Molecules. 2015 May 26;20(6):9671–85. doi: 10.3390/molecules20069671 (PMC6272673; doi:10.3390/molecules20069671)
Supplement: Supplementary file 1 [file molecules-20-09671-s001.pdf]

Supplementary Materials

Table S1. Recovery results for the purity determination of 4-demethylpodophyllotoxin by qNMR.

Analyte	DMPD (mg)	IS (mg)	Molar Ratio Prepared	Assay (%)	Recovery (%)
Set-1	5.94	1.29	0.957	98.9	100.9
Set-1	5.77	1.25	0.960	97.3	99.3
Set-1	6.67	1.38	1.914	98.7	100.7
Set-2	9.85	1.07	1.879	97.8	99.8
Set-2	10.00	1.09	1.907	98.3	100.3
Set-2	11.13	1.16	1.995	98.6	100.6
Set-3	15.03	1.06	2.948	97.9	99.9
Set-3	17.10	1.18	3.012	98.1	100.1
Set-3	15.64	1.09	2.983	98.3	100.3
Mean				98.2	100.2
RSD (%)				0.51	

Table S2. Intra-assay and intermediate precision results for the purity determination of 4-demethylpodophyllotoxin by qNMR.

Analyte	Intra-Assay Precision				Intermediate Precision			
	DMPD (mg)	IS (mg)	Obtained DMPD (mg)	Assay (%)	DMPD (mg)	IS (mg)	Obtained DMPD (mg)	Assay (%)
1	5.94	1.29	5.84	98.3	6.04	1.32	5.93	98.2
2	6.02	1.34	5.91	98.2	6.01	1.30	5.91	98.3
3	5.87	1.30	5.73	97.6	5.85	1.29	5.71	97.6
4	5.96	1.33	5.84	98.0	5.86	1.31	5.75	98.1
5	6.07	1.34	5.95	98.0	6.07	1.36	5.95	98.0
6	6.01	1.30	5.86	97.5	6.15	1.34	5.99	97.4
Mean				97.9				97.9
RSD (%)				0.32				0.36

Table S3. Stability results for the purity determination of 4-demethylpodophyllotoxin by qNMR.

Time (h)	Obtained DMPD (mg)	Assay (%)	Difference (%)
0	5.82	97.8	0.2
8	5.84	98.2	0.2
16	5.82	97.8	0.2
24	5.86	98.5	0.5
Mean		98.1	
RSD (%)		0.34	

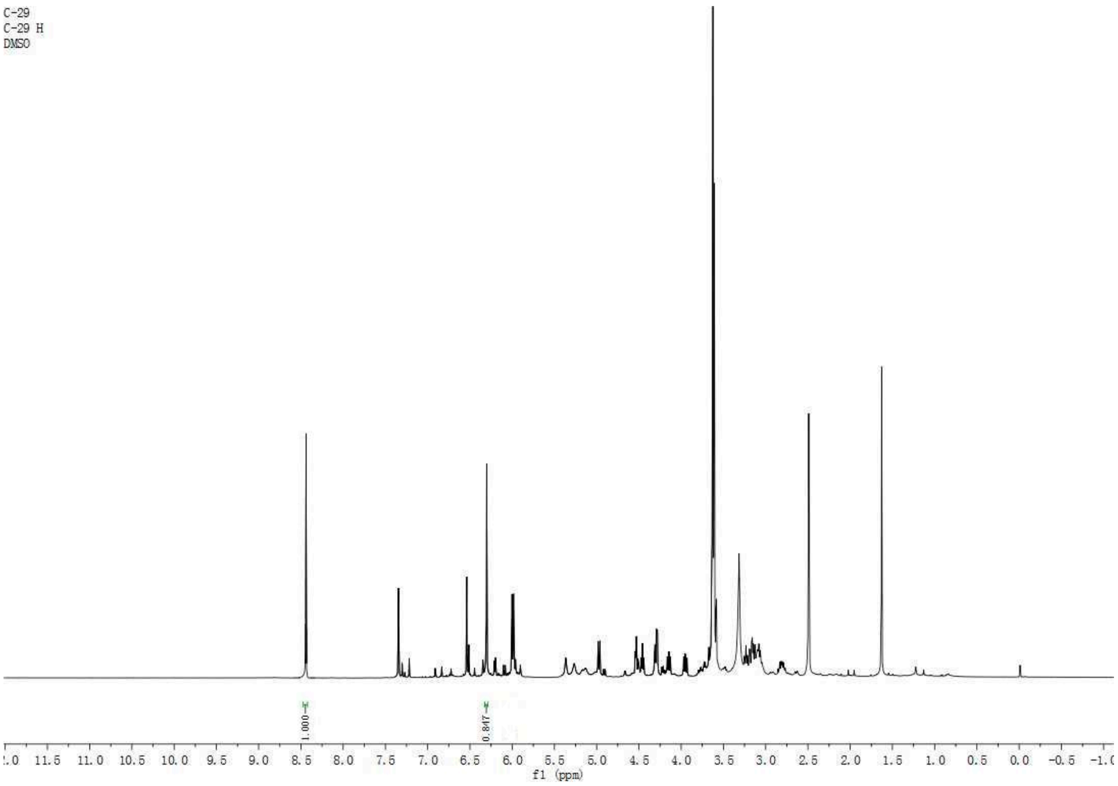

Figure S1. ^{1}H-NMR (500 MHz, DMSO-d_6) spectrum of PDAG.

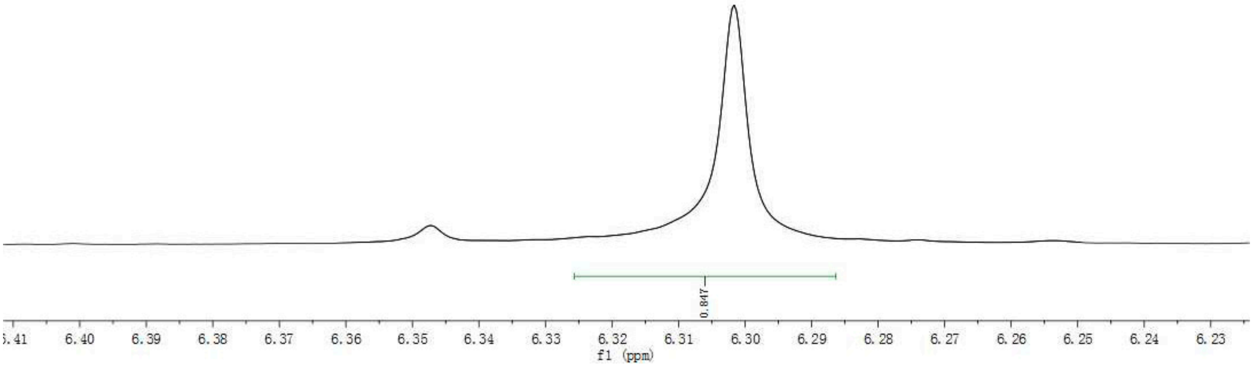

Figure S2. Local amplification of ^{1}H-NMR (500 MHz, DMSO-d_6) spectrum of PDAG.

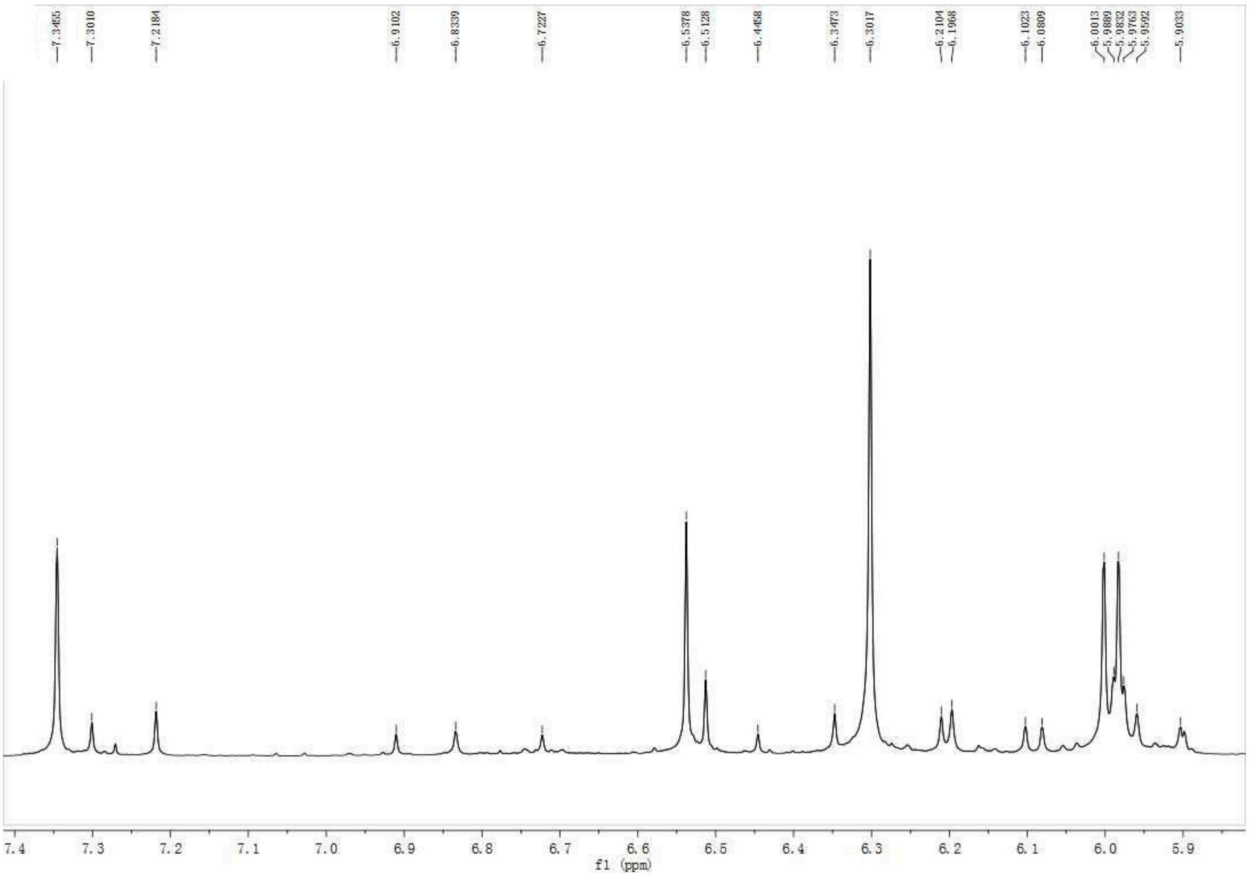

Figure S3. Local amplification of ^{1}H-NMR (500 MHz, DMSO-d_6) spectrum of PDAG.

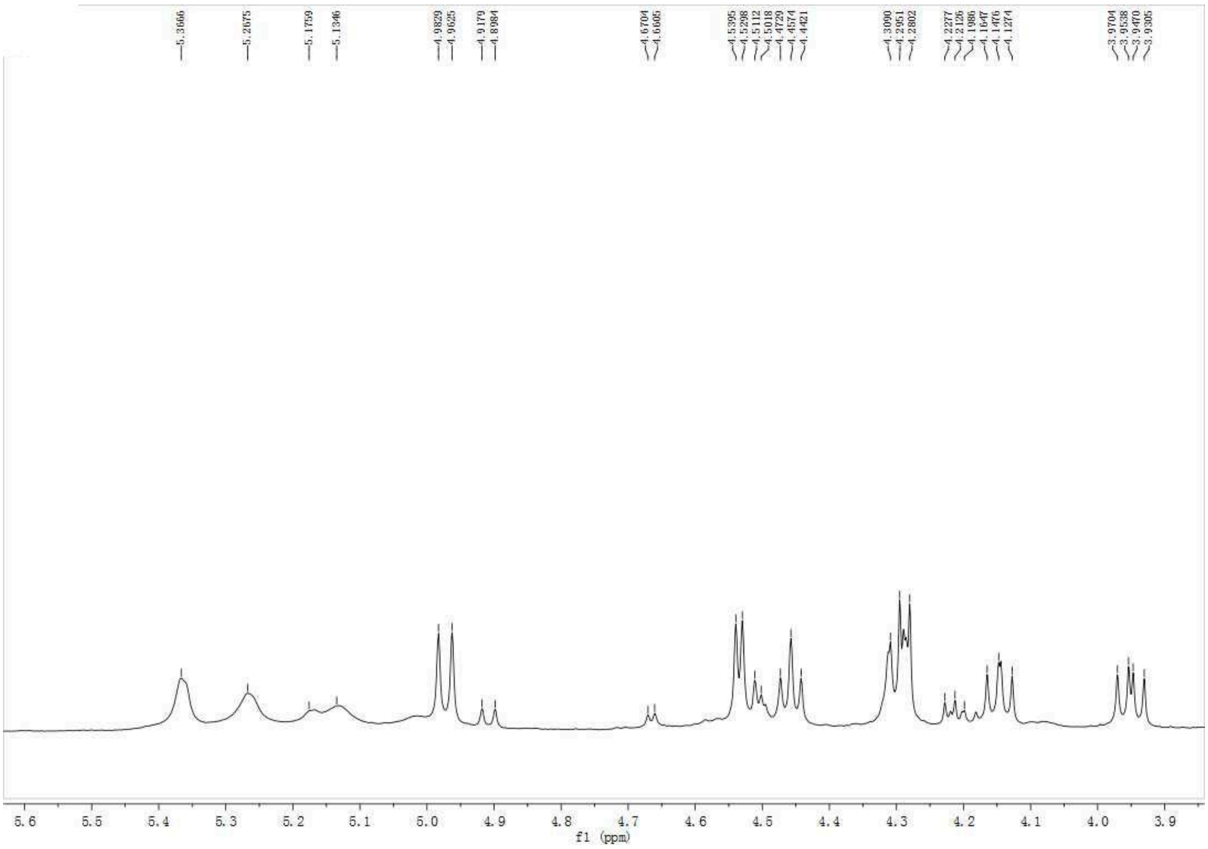

Figure S4. Local amplification of ^{1}H-NMR (500 MHz, DMSO-d_6) spectrum of PDAG.

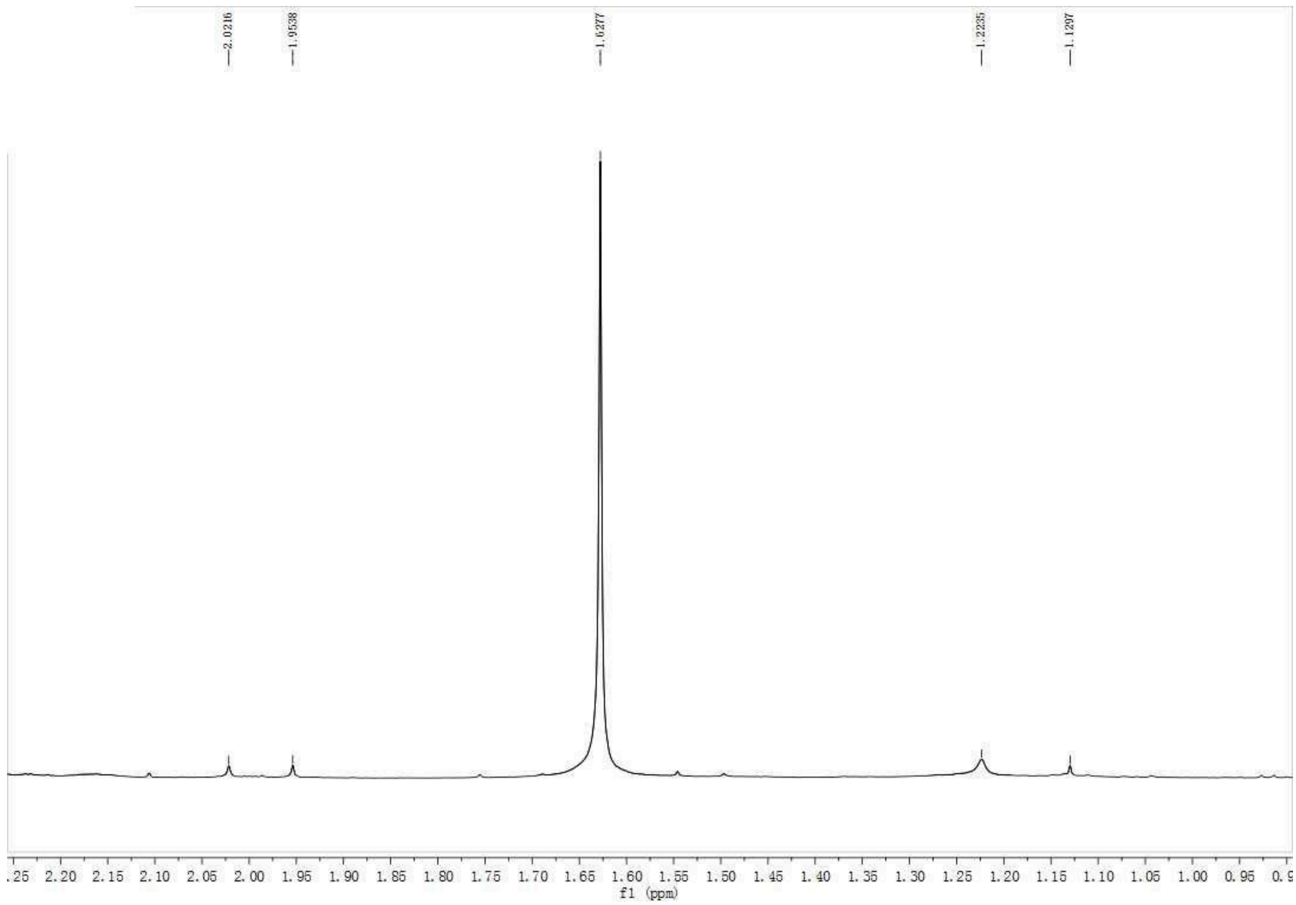

Figure S5. Local amplification of ^{1}H-NMR (500 MHz, DMSO-d_6) spectrum of PDAG.

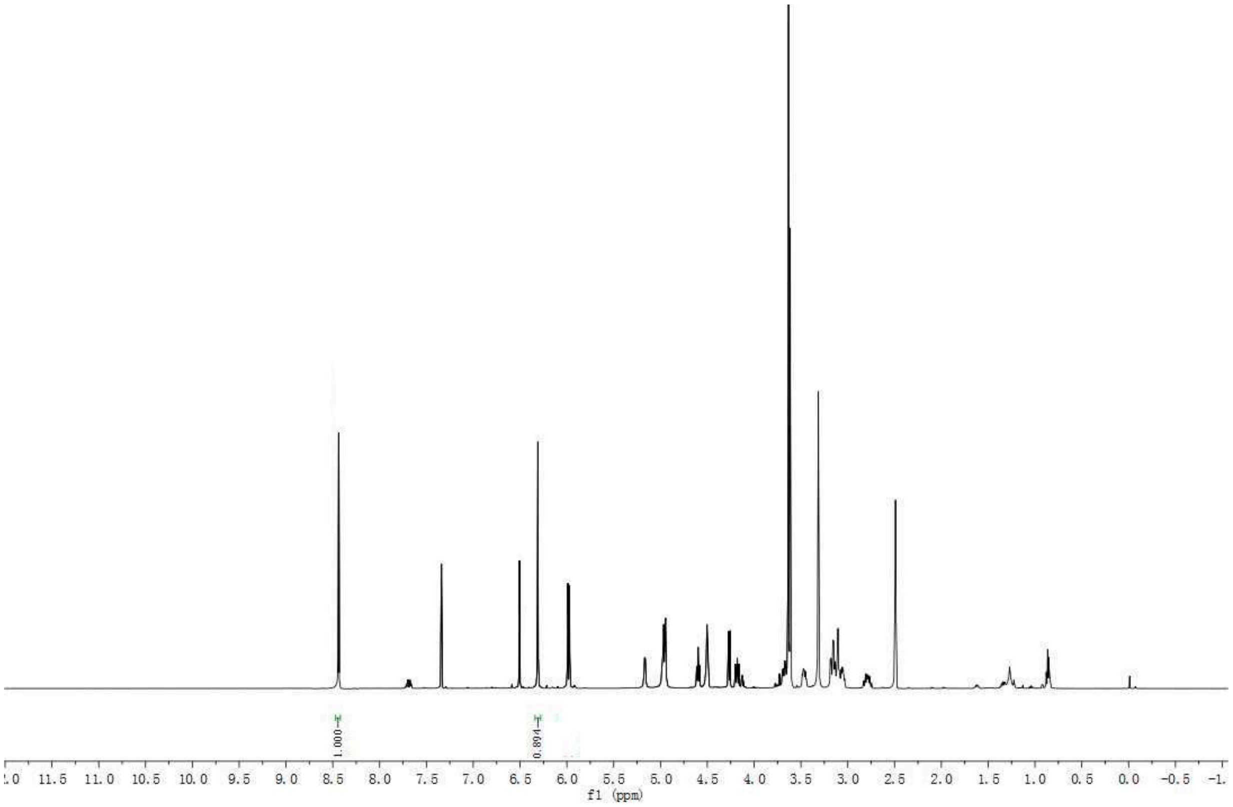

Figure S6. ^{1}H-NMR (500 MHz, DMSO-d_6) spectrum of PDG.

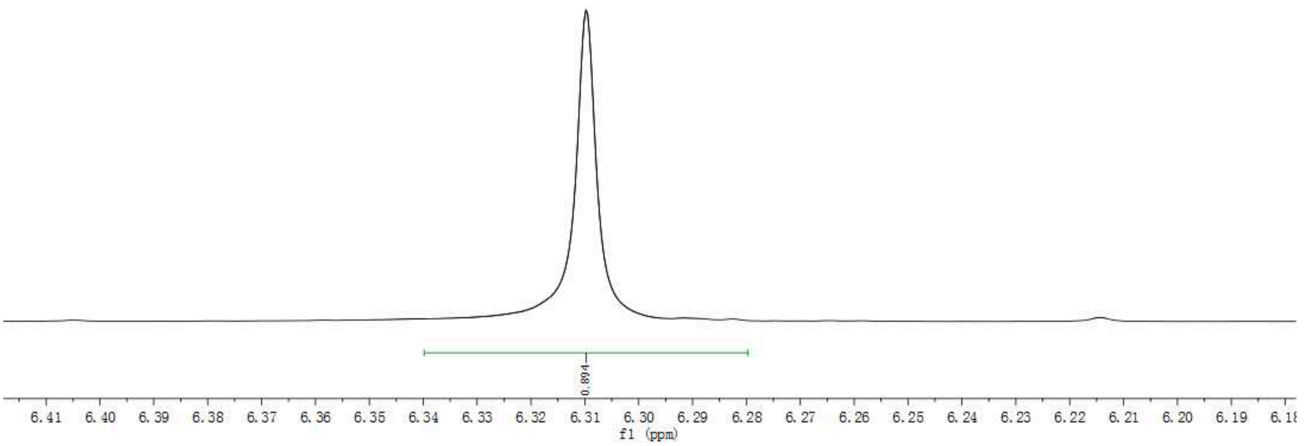

Figure S7. Local amplification of ^{1}H-NMR (500 MHz, DMSO-d_6) spectrum of PDG

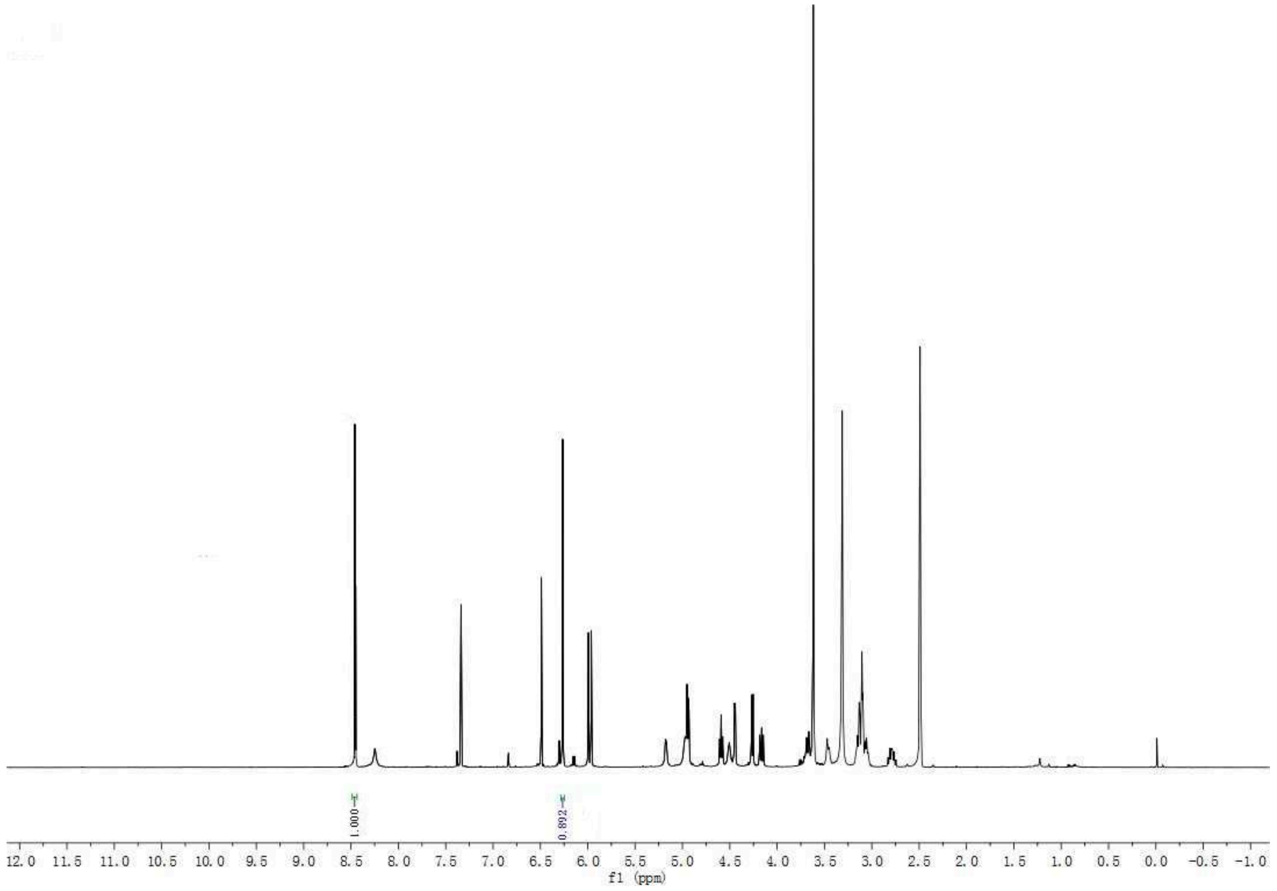

Figure S8. ^{1}H-NMR (500 MHz, DMSO-d_6) spectrum of DMPDG.

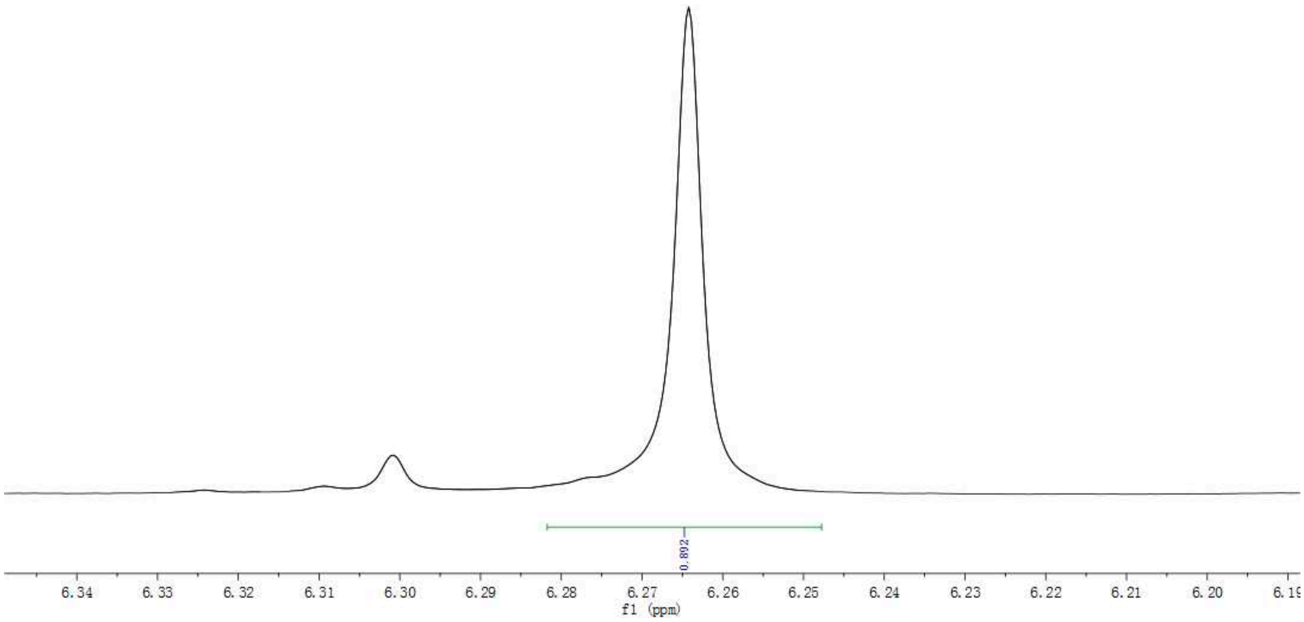

Figure S9. Local amplification of ^{1}H-NMR (500 MHz, DMSO-d_6) spectrum of DMPDG.

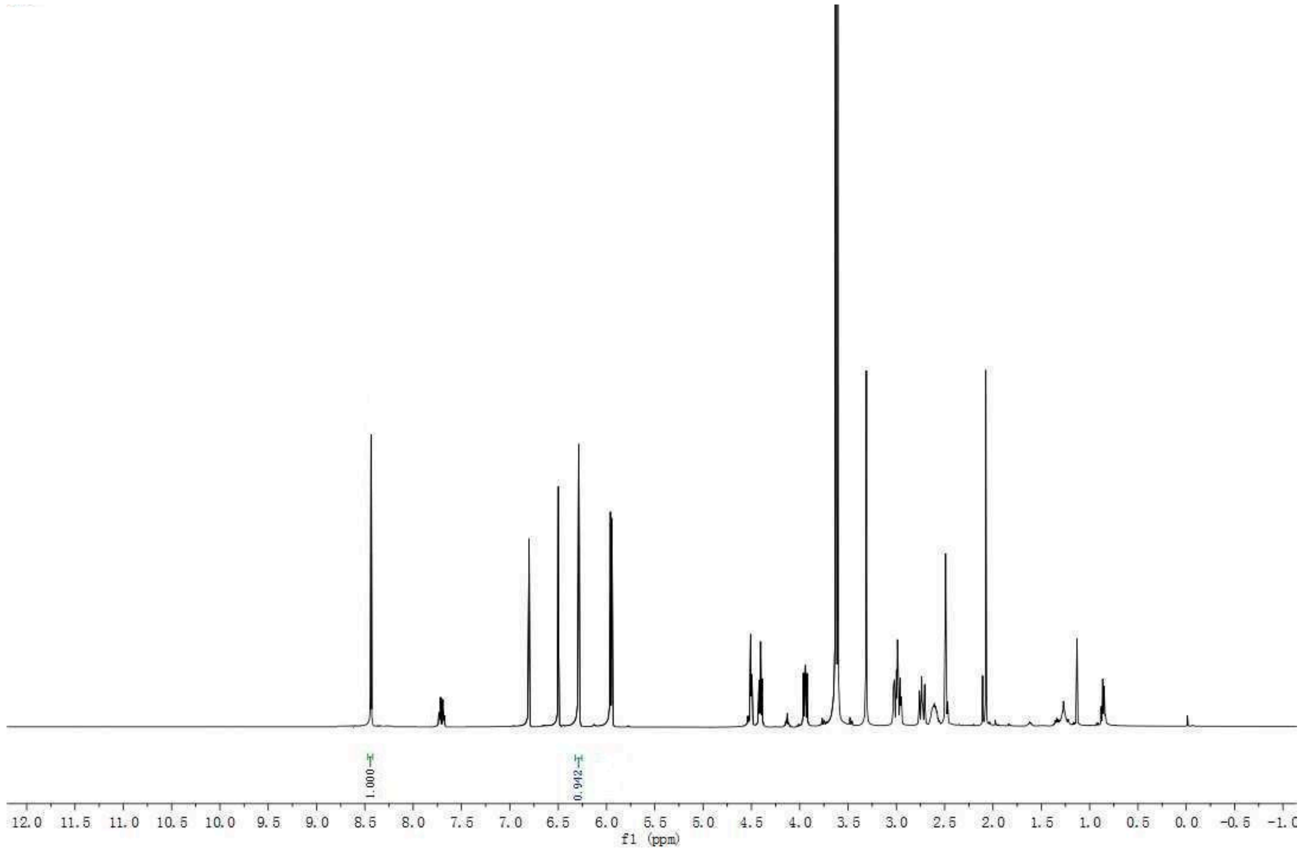

Figure S10. ^{1}H-NMR (500 MHz, DMSO-d_6) spectrum of DPD.

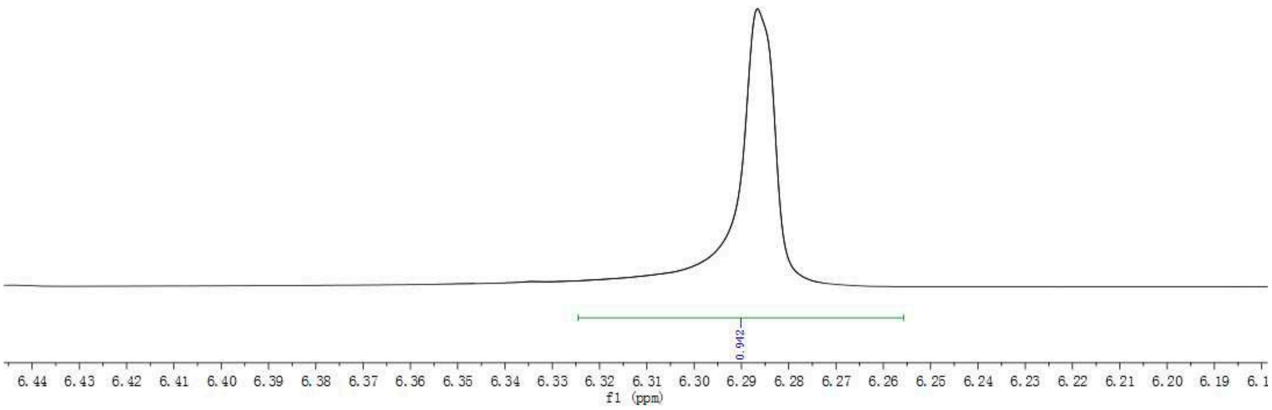

Figure S11. Local amplification of ^{1}H-NMR (500 MHz, DMSO-d_6) spectrum of DPD.

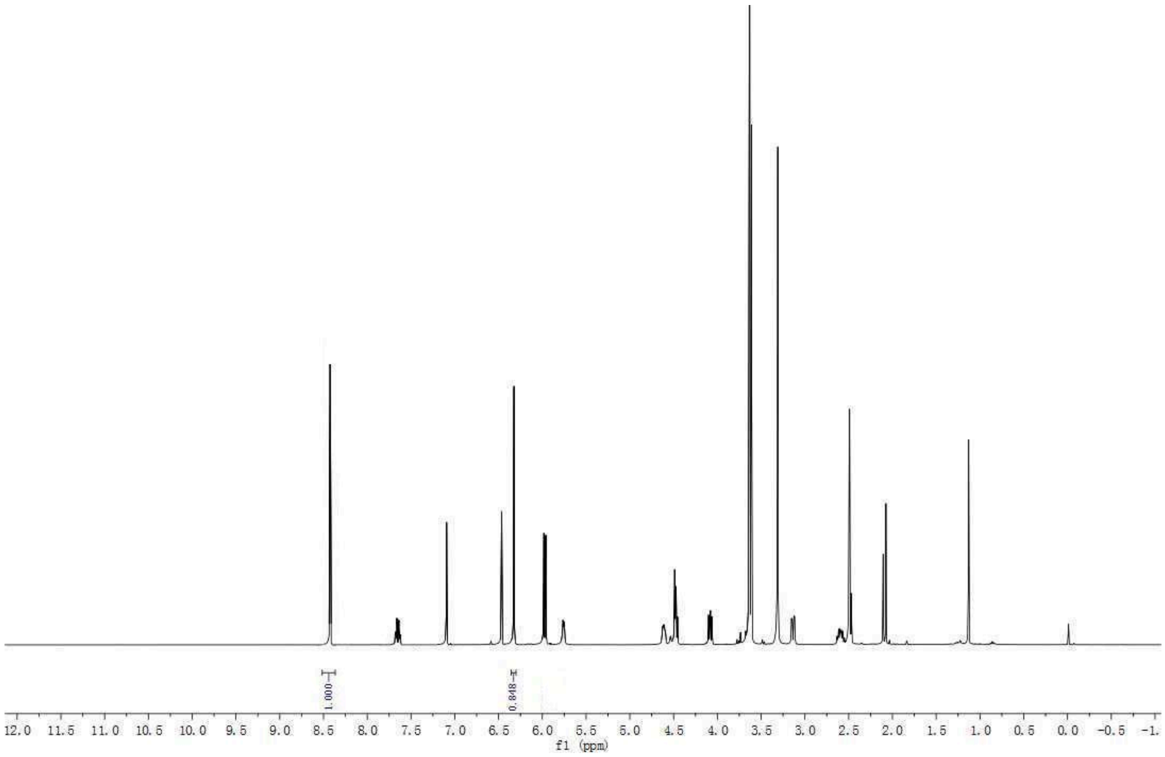

Figure S12. ^{1}H-NMR (500 MHz, DMSO-d_6) spectrum of PD.

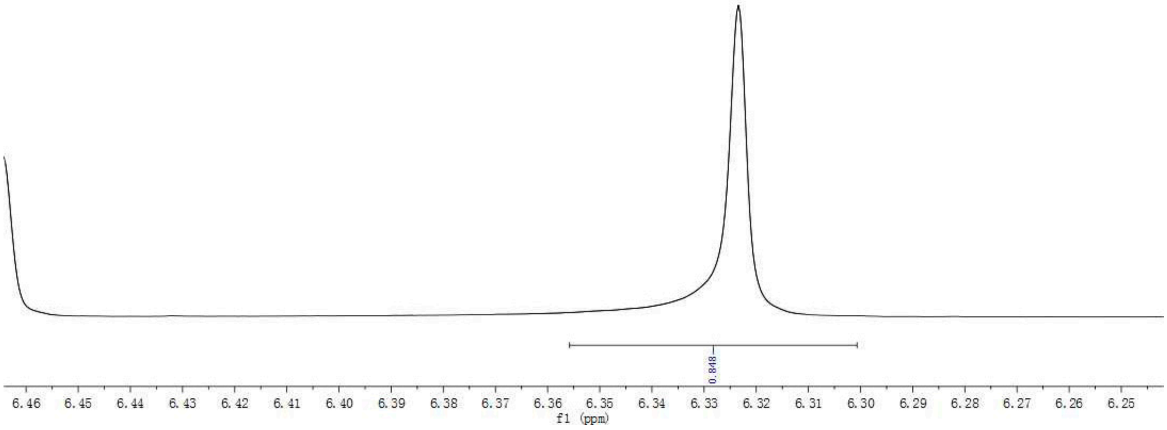

Figure S13. Local amplification of ^{1}H-NMR (500 MHz, DMSO-d_6) spectrum of PD.

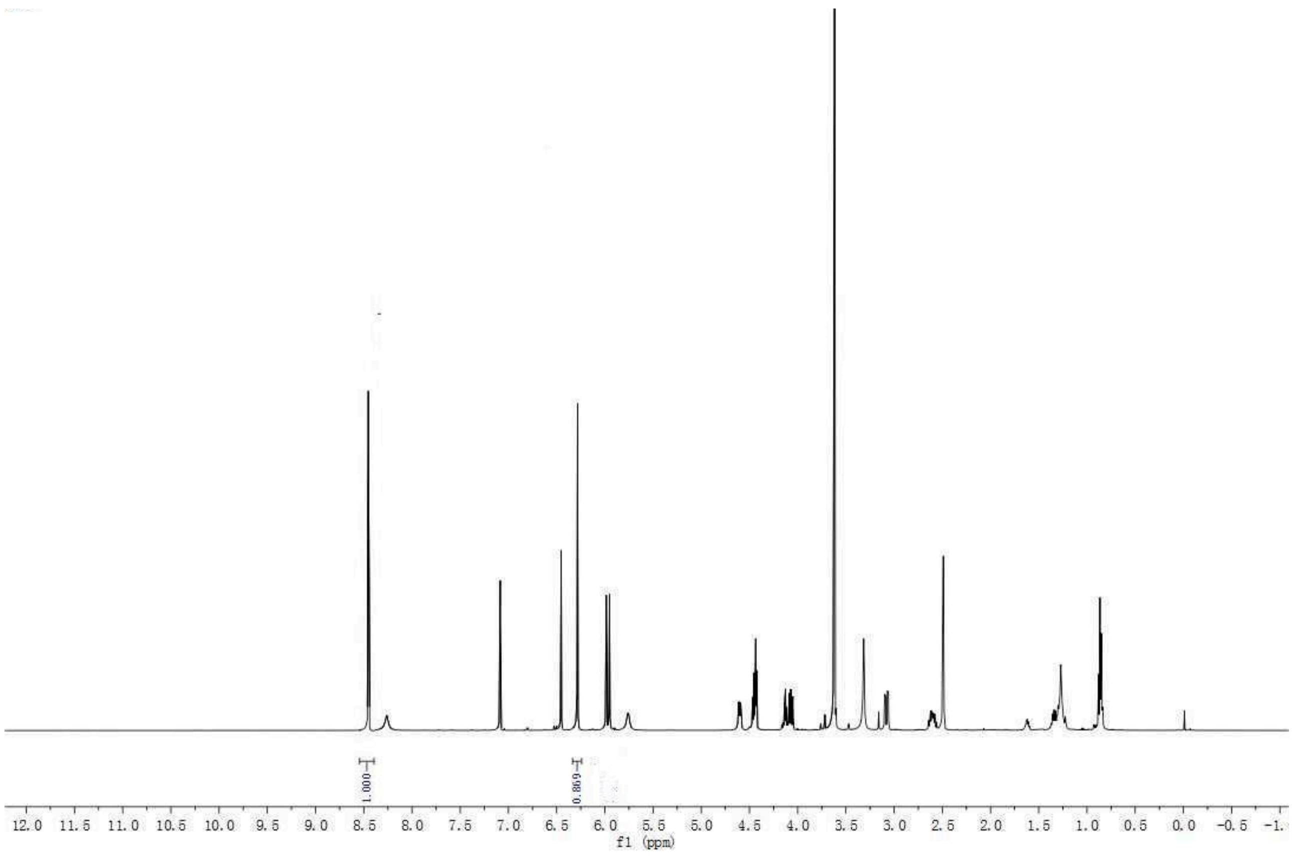

Figure S14. ^{1}H-NMR (500 MHz, DMSO-d_6) spectrum of DMPD.

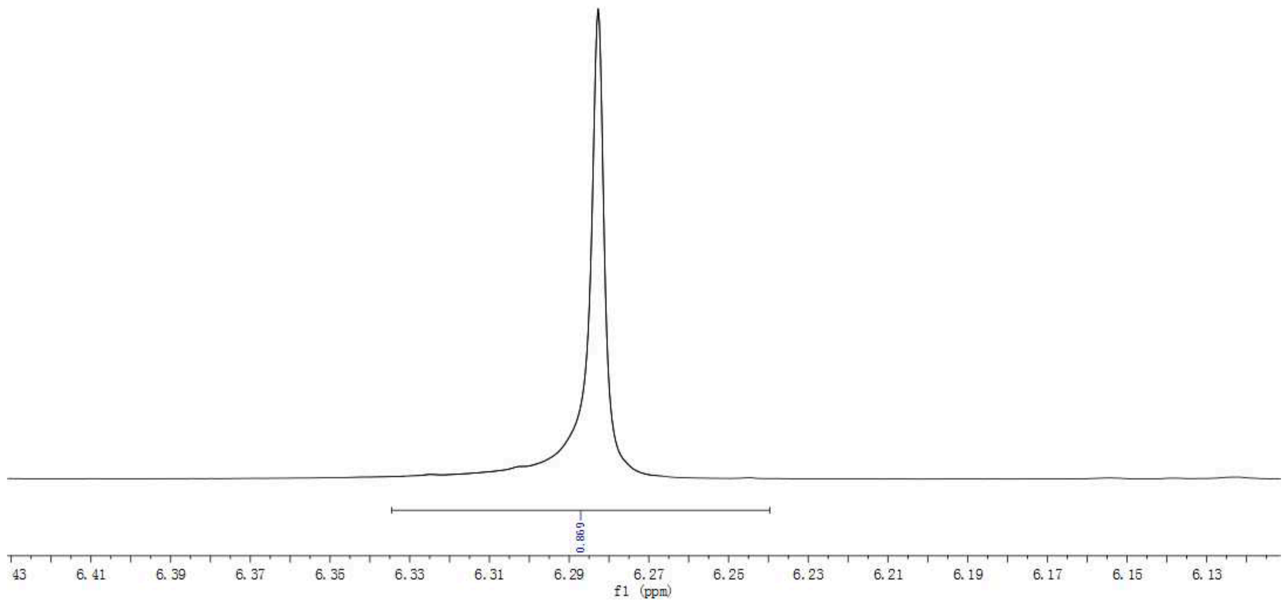

Figure S15. Local amplification of ^{1}H-NMR (500 MHz, DMSO-d_6) spectrum of DMPD.

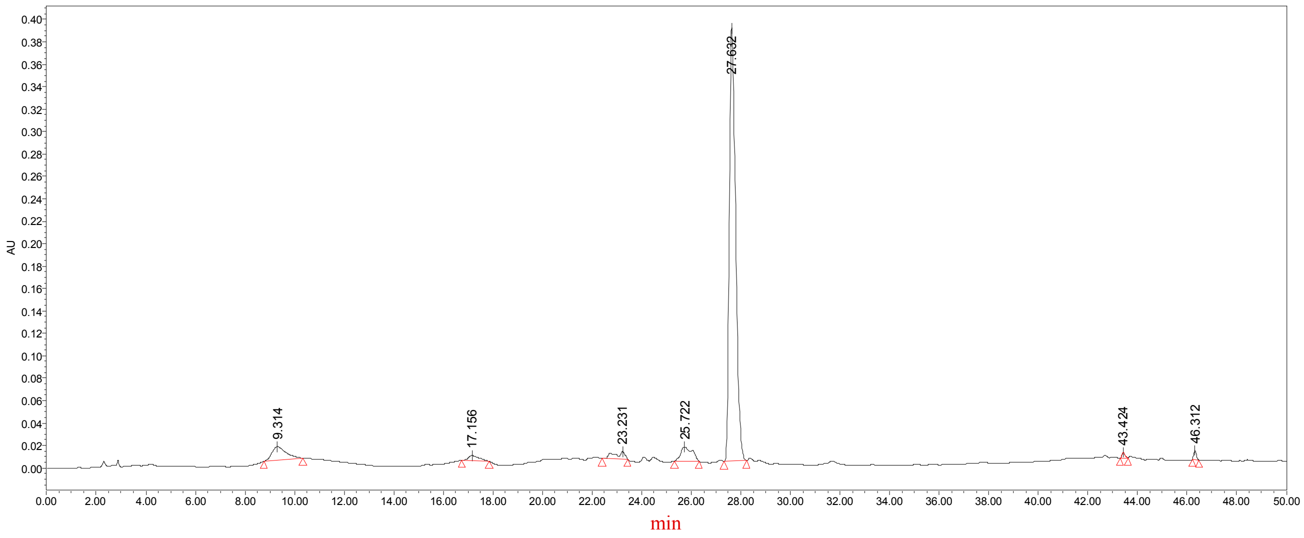

Figure S16. Representative HPLC chromatogram of PDAG.

Table S4. Representative HPLC analysis report of PDAG.

	Time	Area	% Area
1	9.314	149352	2.01
2	17.156	109732	1.48
3	23.231	139201	1.87
4	25.722	180784	2.43
5	27.632	6819176	91.69
6	43.424	9001	0.12
7	46.312	30195	0.41

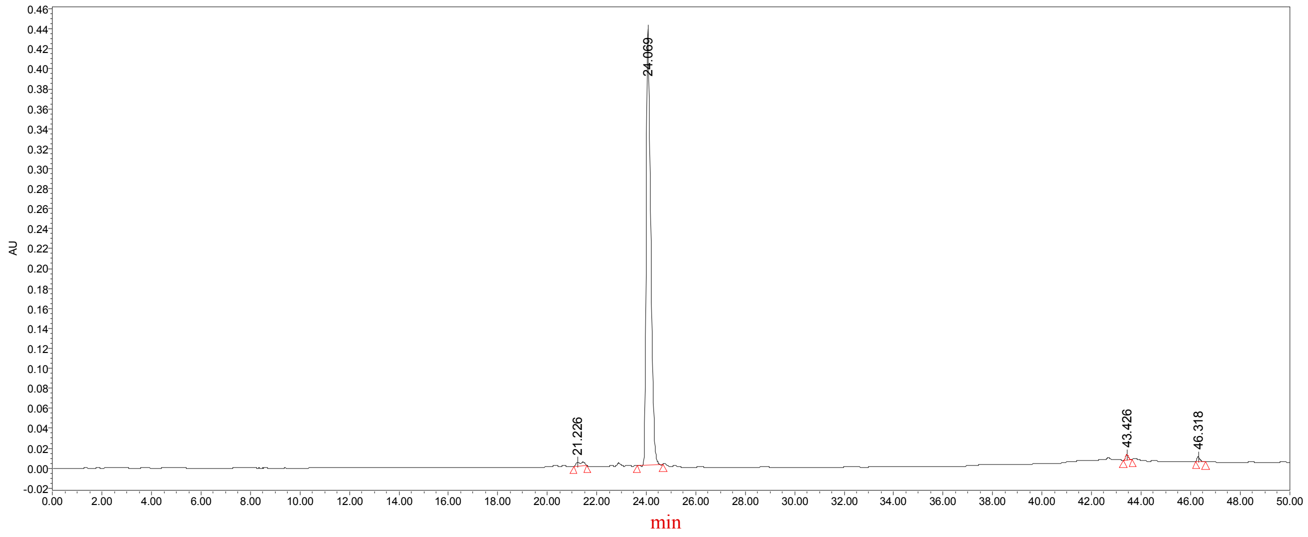

Figure S17. Representative HPLC chromatogram of PDG.

Table S5. Representative HPLC analysis report of PDG.

	Time	Area	% Area
1	21.226	109019	1.90
2	24.069	5540990	96.61
3	43.426	42328	0.74
4	46.318	43111	0.75

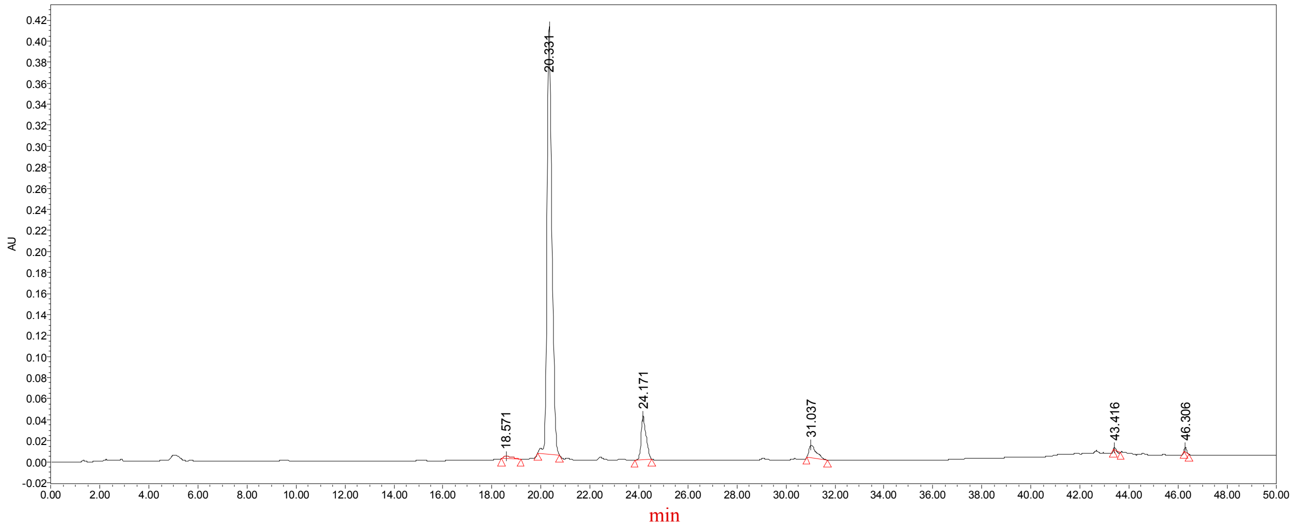

Figure S18. Representative HPLC chromatogram of DMPDG.

Table S6. Representative HPLC analysis report of DMPDG.

	Time	Area	%Area
1	18.571	12864	0.20
2	20.331	6100407	94.53
3	24.171	205992	3.19
4	31.037	73974	1.25
5	43.416	14802	0.23
6	46.306	45051	0.70

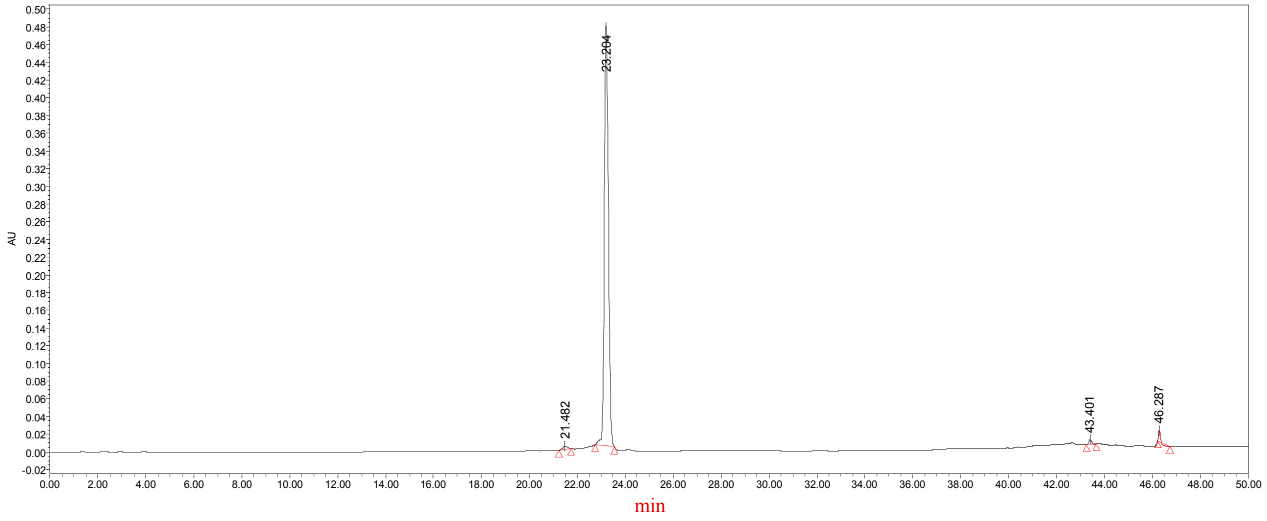

Figure S19. Representative HPLC chromatogram of DMPD.

Table S7. Representative HPLC analysis report of DMPD.

	Time	Area	% Area
1	21.482	63164	1.04
2	23.204	5855114	96.67
3	43.401	28511	1.81
4	46.287	109796	2.28

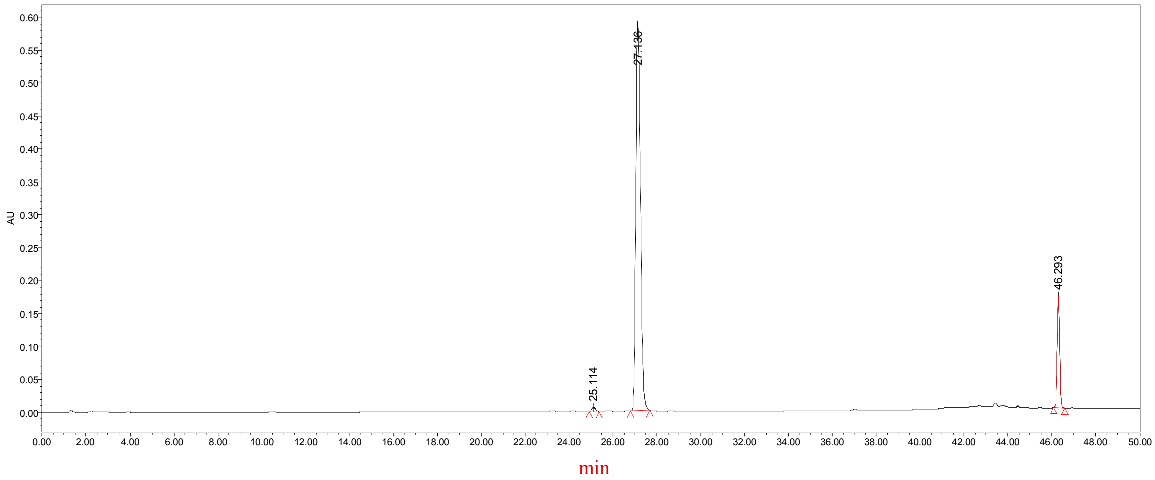

Figure S20. Representative HPLC chromatogram of PD.

Table S8. Representative HPLC analysis report of PD.

	Time	Area	% Area
1	25.114	75543	0.61
2	27.136	11088346	89.91
3	46.293	1169272	9.48

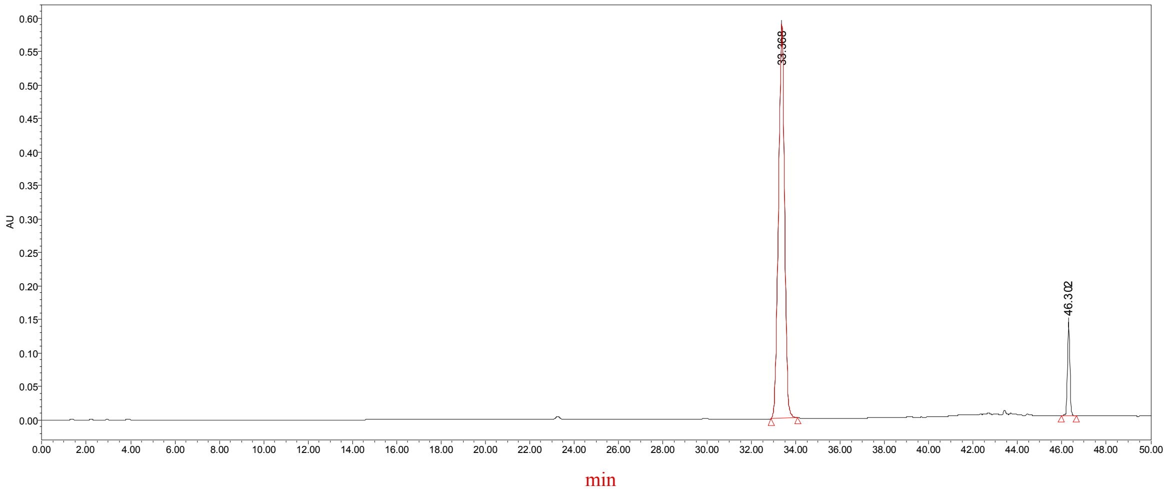

Figure S21. HPLC chromatogram of DPD.

Table S9. Representative HPLC analysis report of DPD.

	Time	Area	% Area
1	33.368	11245395	90.72
2	46.302	1150257	9.28
